# Highly Sensitive Homogeneous Immunoassays Based on Construction of Silver Triangular Nanoplates-Quantum Dots FRET System

**DOI:** 10.1038/srep26534

**Published:** 2016-05-20

**Authors:** Qinghui Zeng, Qin Li, Wenyu Ji, Xue Bin, Jie Song

**Affiliations:** 1 State Key Laboratory of Luminescence and Applications, Changchun Institute of Optics, Fine Mechanics and Physics, Chinese Academy of Sciences, Dong_Nanhu Road 3888, Changchun 130033, P. R. China; 2 Queensland Micro- and Nanotechnology Centre & Environmental Engineering, Griffith University, Brisbane, QLD 4111, Australia; 3 Institute of Nano Biomedicine and Engineering, Department of Instrument Science and Engineering, School of Electronic Information and Electrical Engineering, Shanghai Jiao Tong University, 800 Dongchuan Road, Shanghai 200240, PR China

## Abstract

With growing concerns about health issues worldwide, elegant sensors with high sensitivity and specificity for virus/antigens (Ag) detection are urgent to be developed. Homogeneous immunoassays (HIA) are an important technique with the advantages of small sample volumes requirement and pretreatment-free process. HIA are becoming more favorable for the medical diagnosis and disease surveillance than heterogeneous immunoassays. An important subset of HIA relies on the effect of fluorescence resonance energy transfer (FRET) via a donor-acceptor (D–A) platform, e.g., quantum dots (QDs) donor based FRET system. Being an excellent plasmonic material, silver triangular nanoplates (STNPs) have unique advantages in displaying surface plasmon resonance in the visible to near infrared spectral region, which make them a better acceptor for pairing with QDs in a FRET-based sensing system. However, the reported STNPs generally exhibited broad size distributions, which would greatly restrict their application as HIA acceptor for high detection sensitivity and specificity purpose. In this work, uniform STNPs and red-emitting QDs are firstly applied to construct FRET nanoplatform in the advanced HIA and further be exploited for analyzing virus Ag. The uniform STNPs/QDs nanoplatform based medical sensor provides a straightforward and highly sensitive method for Ag analysis in homogeneous form.

With the requirement of strengthening the global security and health problems, highly selective and sensitive virus antigen (Ag) sensors have become one of the topical subjects in the scientific community. Quantum dots (QDs) donor based fluorescence resonance energy transfer (FRET) by constructing a donor-acceptor (D–A) nanoplatform in aqueous system is becoming increasingly favourable for the medical diagnosis and disease surveillance due to the inherent advantages of QDs^1^,^2^,^3^,^4^,^5^,^6^,^7^. They are particularly advantageous when used for the homogeneous immunoassays (HIA). The increasingly stringent demands of high sensitivity, fast detection, easy steps, and affordable price make FRET based HIA preferable to the heterogeneous immunoassays^1^,^4^,^8^,^9^. Besides, since generally it does not require separation and purification procedures, HIA method often avoids the reduction of the specificity of the biological molecules, hence, better detection sensitivity^9^.

In order to improve the detection sensitivity of FRET-based HIA, researchers need to increase the FRET efficiency generally via increasing the spectral overlap range between acceptor’s absorption and donor’s emission, and/or enhancing the acceptor’s extinction coefficient^1^,^10^. Traditional fluorescent dyes suffer from drawbacks of the self-quenching and inactivation effect, therefore become less and less favorable in the application of FRET based HIA^1^. Quantum dots, due to their unique advantages, are highly suitable for biological labeling and medical immunoassays induced by the variation of fluorescent intensity^1^,^2^,^3^,^4^,^5^,^6^,^7^, and have already been a top choice as a donor candidate for FRET via D–A system. Among the QDs, the red-emitting QDs are preferable because of less interference from autofluorescence background caused by biomolecules, deeper penetrate depth of biological tissue and no need of relative longer excitation wavelength light source requirement.

From the aspect of acceptor, similar to gold nanoparticles, silver nanoparticles with strong plasmon absorption property appear to be better energy acceptors because of higher extinction coefficient, etc. However, most of the plasmon absorption peaks of silver nanoparticles are located at the near ultraviolet or purple window^11^,^12^,^13^,^14^, which separates far away from most QDs’ emission window, let alone the red emission window. Because of nanoplates’ in face oscillations of conduction electrons, silver triangular nanoplates (STNPs) could display surface plasmon resonance in the visible to near infrared spectral region. STNPs should thus serve as a better acceptor because of their high extinction coefficient, optical and chemical stability. At the same time, their absorption and scattering bands could be tuned into red-light wavelength by adjusting the size of the STNPs^15^,^16^,^17^,^18^. This red plasmon resonance absorption band can match well with the emission window of CdTe QDs, which have been generally used in aqueous system. However, the STNPs prepared according to the previous method are often difficult to obtain high uniformity in size and morphology, which will potentially restrict their application as HIA acceptor to meet the demand of high detection sensitivity and repeatability, let alone in real world applications in the future.

In order to solve this problem, we have prepared the relatively uniform STNPs with homogeneous morphologies, size, and narrow size distribution via tuning the concentration of OH^−^ ions (Figure S1 in the Supporting Information), which exhibit strong plasmon absorption peaks^19^. QDs’ emission and STNPs’ plasmon absorption were size-adjusted to ensure an high FRET efficiency by optimizing the spectral overlap range between the emission band of QDs and absorption band of the STNPs^16^,^17^. We further improved our sensor design based on the reported prototype^1^,^20^ and demonstrated the QDs-STNPs based FRET sensor for detection of Hepatitis B surface antigen (HBsAg). After the construction of the sandwich structure nanoplatform shown in Fig. 1, the photoluminescence (PL) of QDs was quenched by the STNPs instantly. The HBsAg was detected synchronously against the HIA to confirm the detection range, detection sensitivity and detection specificity. As far as we know, this work provides the first report on employing QDs as donors and STNPs as acceptors to construct FRET platform for HIA of virus antigen.

## Results and Discussion

The improved synthesis of the STNPs was a seed-mediated growth method according to our previous method^19^. Firstly, silver salts were reduced with a strong reducing agent to prepare the seed particles. Afterwards, the silver seed particles were immediately irradiated by a sodium lamp (70 Watt) for 2 hours. Briefly, the triangular nanoplates with plasmon absorption peak at 613 nm were synthesized by using citrate acid as surface ligand^19^,^21^. Figure S1 in the Supporting Information expounds the growth process of the highly uniform and nonuniform STNPs schematically. The highly uniform STNPs (see the FESEM in Fig. 2) were prepared by the additional control of the OH^−^. As shown in Figure S2 in the Supporting Information, the low magnification of the FESEM and the narrow size distribution of the STNPs prove well the high quality and high uniform of the prepared STNPs. Without the addition of OH^−^, the resulting STNPs present heterogeneous morphologies, size and broad size distribution (see the FESEM in Figure S3), proved by the widening of the full width at half maximum (fwhm) of the plasmon resonance absorption spectrum (Figure S3 in the Supporting Information). The fwhm value can be decreased obviously from 117 nm to 83 nm after the introducing of the OH^−^. This is because of the fact that introduction of OH^−^ could evenly inhibit the generation rate of silver ions in sum^19^. We indeed observed that the oxidation of silver nanoparticles was evidently restrained by introducing OH^−^. Though light irradiation will speed up the oxidized process of small silver nanoparticles due to the temperature elevation, OH^−^ ions appear to have effectively inhibited the side-effect. Therefore, introducing OH^−^ can inhibit the oxidization of silver nanoparticles and slow down the generation rates of silver ions, which is favourable for kinetic control growth^19^,^21^. As shown in Fig. 2, the length of side of the triangular nanoplates is about 70 nm. In our case, the size-tuned red QDs (λ_em_ = 612 nm) were selected to be the donor for the HIA because they match well with the localized plasmon resonance absorption band of the STNPs.

Since the QDs and STNPs were capped with negatively charged 3-mercaptopropionic acid and citric acid ligands, respectively, thus, when the QDs and STNPs were mixed together in the same vessel, they could not combine with each other due to the electrostatic repulsive force. Consequently, the PL of QDs would be kept constant. As shown in Figs 3 and 4a, when the volume of STNPs added into the QDs’ solution was increased from 5 to 10, 20, 40 μL, and upwards, the PL intensity of QDs almost kept constant. However, when the ethylenediamine modified STNPs was added into the QDs’ solution, the QDs would readily combined with the STNPs through the bridge link of ethylenediamine due to the electrostatic interaction. Consequently, the PL of QDs would be efficiently quenched by the STNPs due to the nonradiative FRET process. The digital photos of the QDs’ solution before and after the addition of ethylenediamine modified STNPs under the excitation of the ultraviolet lamp are shown in Figure S4 in the Supporting Information. It can be observed by naked eyes that the QDs’ fluorescence is quenched dramatically after the introduction of the ethylenediamine modified STNPs. As shown in Figs 3 and 4b, when the volume of ethylenediamine modified STNPs added into the QDs’ solution was increased from 5 to 10, 20, 40 μL, and upwards, the PL intensity of QDs was quenched severely and the quenching approached saturation until the volume of STNPs reached about 160 μL.

The FRET efficiency can be estimated by Ф_FRET_ = 1 − I_DA_/I_D_ assuming that other quenching mechanisms, such as re-absorption, are negligible, where I_DA_ and I_D_ are the fluorescent intensity of the FRET donor with and without the presence of the FRET acceptor, respectively^1^,^3^. According to the PL spectra shown in Fig. 4b, the FRET efficiency could achieve 98% for QDs at quenching saturation point. This high FRET efficiency demonstrate a synergetic effect owing to the properties of the present design^3^. Firstly, STNPs have larger surface area and no curvature, which can amplify the quenching sites and increase the quenching efficiency. Secondly, STNPs have a large extinction coefficient. The extinction coefficient of STNPs is about 10^12^ M^−1^cm^−1^ (see the details in the supporting information) which is about 2–3 orders of magnitude higher than that of gold nanoparticles and even 7–8 orders of magnitude higher than that of fluorescent dyes. Thirdly, the spectra of the STNPs and QDs overlap ideally owing to the rational tunability of the spectra of our tailor-designed QDs donor and STNPs acceptor. Obviously, the more the spectra overlap between donor and acceptor is, the higher the FRET efficiency would be. Finally, the absorption of STNPs at the excitation of the donor, e.g., 400 nm for QDs is low, which will eliminate as much as possible the adverse impact of the FRET effect. Because the PL quenching of QDs is correlated to the STNPs’ dosage, we could perform the HIA based on the quenching of QDs’ fluorescence when the highly efficient and robust QDs were function as the detection tracer agent.

**Figure 1:**
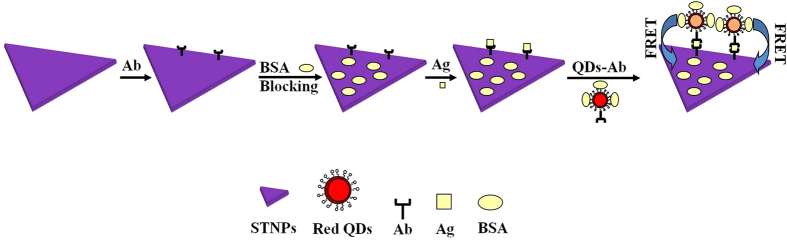
The schematic immune detection route of antigen based on the construction of FRET sandwich structure nanoplatform from QDs to STNPs.

**Figure 2:**
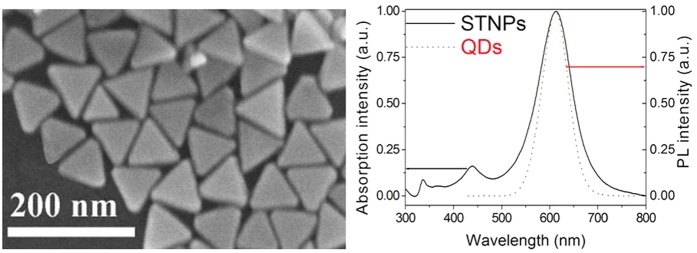
FE-SEM image of the STNPs prepared in our method and the normalized absorption spectrum of the STNPs and the PL spectrum of QDs. The scale bar is 200 nm.

**Figure 3:**
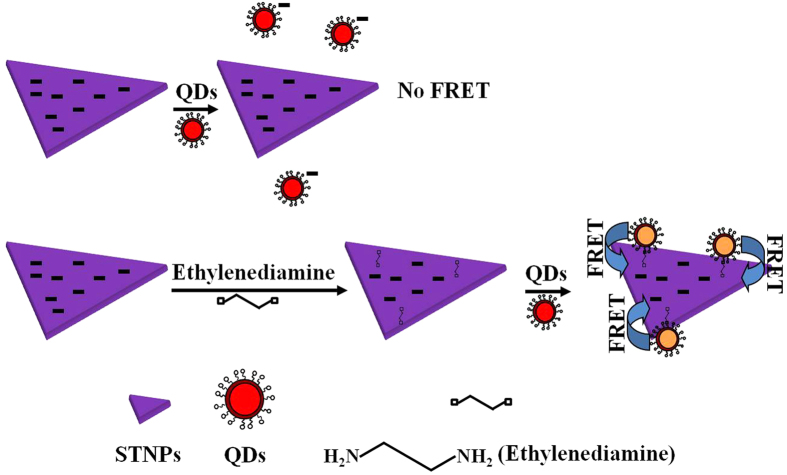
The schematic quenching mechanism based on the FRET from QDs to ethylenediamine modified STNPs.

**Figure 4:**
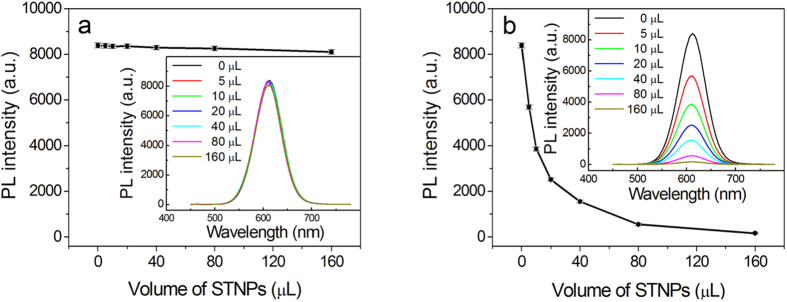
The variation of the PL intensity at the QDs’ emission peak after addition of different volumes of STNPs (**a**) and ethylenediamine modified STNPs (**b**). The volume of STNPs was increased from 5 to 10, 20, 40, 80 and 160 μL. The concentration of QDs was fixed as 14 nM. Inset is the corresponding PL spectra evolution of QDs.

In this work, Hepatitis B virus was selected to carry out HIA based on the QDs-STNPs FRET nanoplatform. The schematic HIA route of HBsAg is given in Fig. 1. STNPs immobilized with HBsAb1 on the surfaces, are employed as FRET acceptors. On the other hand, the red-emitting QDs (λ_em_ = 612 nm) immobilized with HBsAb2 through the covalent binding (Figure S5 in the Supporting Information), are employed as FRET donors. Addition of analyte HBsAg to the HIA solution triggered the immune reactions induced sandwich construction and resulting in an Ag concentration-correlated FRET processes. Before the detection test, the STNPs-Ab1 and QDs-Ab2 were firstly mixed together in a homogeneous solution. When the analyte Ag was added into the solution, the STNPs-Ab1/Ag/QDs-Ab2 sandwich structure would form due to the immune reactions and the FRET from QDs to STNPs would take place spontaneously.

**Figure 5:**
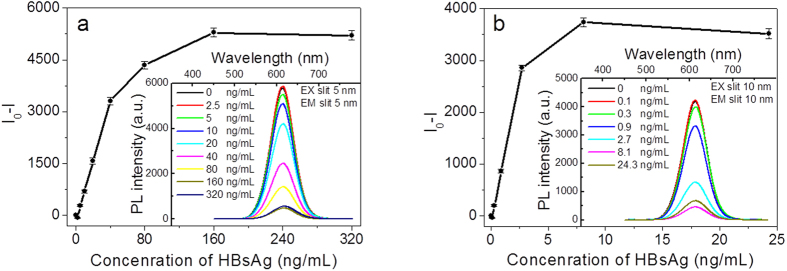
FRET-based detection of antigen with supplied QDs-Ab concentration of 7 nM (**a**) and 0.35 nM (**b**). The relationship between the subtracted PL intensity (I_0_−I) of the QDs-HBsAb in the presence of STNPs-Ab1 and the detected HBsAg with different concentrations. Inset is the corresponding PL spectra variation of the QDs.

**Figure 6:**
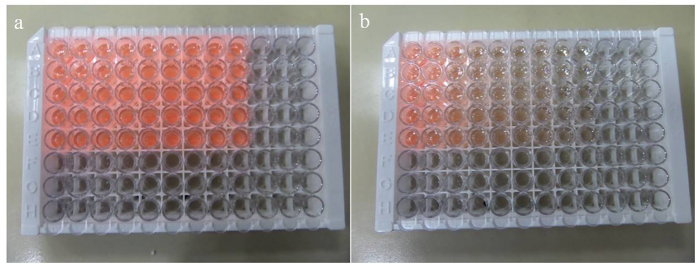
The digital photo of the QDs’ kit before (**a**) and after (**b**) FRET-based detection of antigen under the excitation of the ultraviolet lamp. From left to right, the concentration of the detected antigen is increased from 0 to 2.5, 5, 10, 20, 40, 80, 160, and 320 ng/ml. Each antigen concentration is detected by the statistics of five parallel samples.

**Figure 7:**
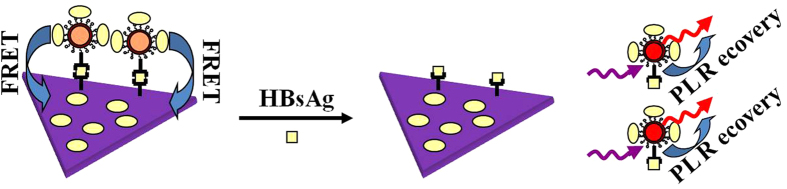
The schematic fluorescence recovery mechanism caused by the supersaturated detected Ag.

As shown in the inset of Fig. 5 and digital photo of the QDs’ kit in Fig. 6, the PL quenching exhibits an evolving FRET process *vs.* the increase of the concentration of HBsAg. The detection curves were obtained by the subtraction between PL intensity of the control sample of QDs-Ab2 without Ag and that of STNPs-Ab1/Ag/QDs-Ab2 sandwich structures with Ag. Figure 5a,b illustrate the PL spectra of HIA system with different concentrations of antigens when the dosage concentration of QDs-Ab2 was 7 nM and 0.35 nM, respectively. It was observed that the fluorescence intensity increased monotonically with the gradual increase of the Ag’s concentration until 160 ng mL^−1^ for higher QDs’ concentration and 8.1 ng mL^−1^ for lower QDs’ concentration, respectively. Therefore, the detection range of HBsAg can achieve 160 ng mL^−1^ for this HIA. After a linear fit, the linear calibration curve could be acquired as Y = 86.0671X − 148.2 (5 ≤ X ≤ 40 ng mL^−1^, R = 0.997) for higher dosage HBsAg and Y = 1088.7X − 93.96 (0.3 ≤ X ≤ 2.7 ng mL^−1^, R = 0.999) for lower dosage HBsAg detection, respectively. The limit of detection (LOD) have been defined as the lowest concentration of detected sample that gives positive value of the PL intensity subtraction between that of control and detected sample^1^,^22^. It can be distinctly observed from Fig. 5b that the subtracted PL intensity was still discernable to be positive even when the concentrations of HBsAg were as low as about 300 pg mL^−1^ for the HIA when the lower dosage concentration was supplied. The LOD of this HIA can thus achieve as low as 300 pg mL^−1^. As a result, we can perform the extremely accurate detection in a relative wide concentration range and highly sensitive detection requirement (e.g., 300 pg mL^−1^ ~ 160 ng mL^−1^). Herein, the LOD is 27.7 times lower than the previous result by using the gold nanorods (GNRs) as FRET acceptor (8.3 ng mL^−1^)^1^, which is due to the higher FRET efficiency as discussed in the above. Relevant parameters of STNPs and GNRs are shown in Table S1; the comparison explains the reason of the higher FRET efficiency and lower LOD of the antigen detection when using STNPs. In a word, two reasons could not be ignored: firstly, STNPs have larger surface area and no curvature, which can amplify the quenching sites and increase the FRET efficiency. Secondly, STNPs have a larger extinction coefficient compared with that of gold nanoparticles. In theory, when the concentration of the analyte Ag exceeds the saturated concentration of the labelled Ab, e.g., 320 ng mL^−1^ for HBsAg in higher Ag concentration immunoassay and 24.3 ng mL^−1^ for HBsAg in lower Ag concentration immunoassay, there should be no further response for the immunoassay. In fact, there was a little decline in the detection curve. This is because the addition of detected HBsAg to the kit had replaced the sandwich Ag and leading to a fluorescence recovery of the QDs (Fig. 7).

**Figure 8:**
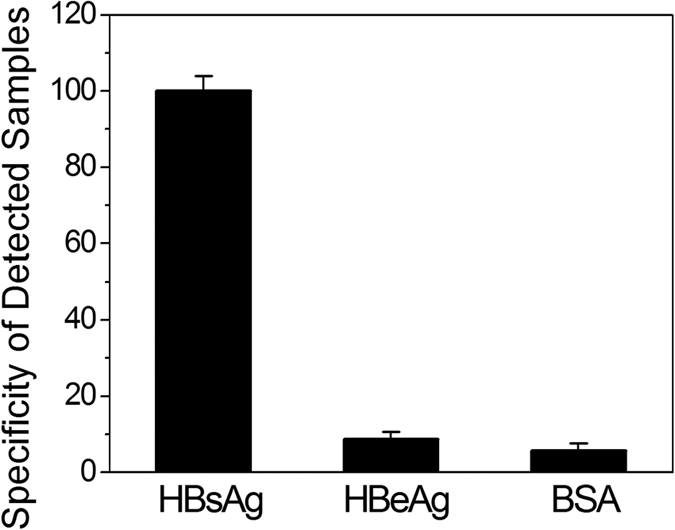
The specificity on the nonspecific binding experiments of BSA (1%) and HBeAg using the FRET based homogeneous immune detection by considering the HBsAg’s specificity as 100%.

The specificity of an immune detection mainly relies on the immunoreactivity of the labelled monoclonal antibodies. In order to prove the high detected specificity of our FRET based HIA, we also performed some non-specific detection. To validate the specificity, a control test was performed by applying a 1% BSA solution as the analyte sample. Because both the structure and morphology properties of HBeAg and HBsAg are similar to each other, it is necessary to detect the HBeAg to further confirm the specificity of the HIA. The results are shown in Fig. 8, in which the fluorescence intensity is normalized to the value of HBsAg detection by considering the HBsAg’s specificity as 100%. When the QDs-HBsAb conjugates are used to detect the nonspecific binding of the HBeAg, a low fluorescence intensity can be observed. This phenomenon implies that there was 8.6% nonspecific binding of HBeAg. Similarly, when the QDs-HBsAb conjugates are used to detect the nonspecific binding of the BSA sample, only a small PL signal is observed under the same detection condition. This phenomenon implies that only 5.6% nonspecificity with BSA molecules occurs. Obviously, the HIA using the QDs-HBsAb conjugates are much specific for HBsAg detection since the specificity is higher than 90%. As a result, our HIA based on the QDs to STNPs FRET nanoplatform are credible and feasible.

## Conclusions

We have prepared the highly uniform STNPs through controlling of the additional OH^−^ ions. Our robust QDs’ emission peaks match well with the tailor-designed STNPs’ absorption spectra to construct a D-A system for highly effective FRET. The FRET efficiency between CdTe QDs and STNPs can reach 98%, chiefly owing to the uniform triangle nanoplate shape of the STNPs, and the superior plasmonic absorption of STNPs with an extinction coefficient 2–3 orders of magnitude higher than that of gold nanoparticles. The HIA of virus antigen is demonstrated by FRET induced quenching of QDs’ fluorescence based on the construction of the sandwich STNPs-Ab1/Ag/QDs-Ab2 nanoplatform. The detection sensitivity for HBsAg is as high as 300 pg mL^−1^, 27 times higher compared to that of the QDs-GNRs FRET systems. The detection range can reach 160 ng mL^−1^ for HBsAg HIA. This STNPs/QDs nanocomposite based nanosensor offers a simple but sensitive approach for antigen detection in a homogeneous format and has the potential to be applied in wide areas with very high sensitivity requirement, such as medical-immune detection and clinical test.

## Methods

### Chemicals and reagents

Silver nitrate (AgNO_3_, ≥99%) was purchased from Fluka. Ethylenediamine (≥98%), sodium borohydride (NaBH_4_, ≥98%), 3-mercaptopropionic acid (MPA, 98%), sulfo-N-hydroxysulfosuccunimide (sulfo-NHS, ≥98.5%), and 1-ethyl-3-(3-dimethylaminopropyl) carbodiimide hydrochloride (EDC, ≥99%) were purchased from Aldrich. Trisodium citrate (≥99%) and sodium hydroxide (NaOH, ≥98%) were purchased from Beijing Chemical Works. HBsAg, Hepatitis B e antigen (HBeAg), HBsAb1, HBsAb2 and bovine serum albumin (BSA) were purchased from H & R Bioscience Co., Ltd. Deionized water was purified by a Milli-Q system and the resistivity was 18.2 MΩ**·**cm.

### Synthesis of silver tranigular nanoplates

The improved synthesis of STNPs was a seed-mediated growth method according to our previous publication^21^. Firstly the silver salt was reduced with a strong reducing agent in water at room temperature, to prepare about 5 nm seed particles. Afterwards, the silver seed particles were immediately irradiated by a sodium lamp (70 Watt) for 2 hours. Simply, 24.25 ml deionized water, AgNO_3_ (250 μL, 10 mM), and trisodium citrate (250 μL, 100 mM) were mixed under vigorously stirring at room temperature. To this mixture, 250 μL mixed aqueous solution (NaBH_4_ (8 mM), NaOH (0.125M)) was injected via dropwise addition. The resulting silver seeds were immediately irradiated by a sodium lamp (70 Watt) for 2 hours. During the irradiation, the reaction was kept stirring all through. To synthesize high uniform STNPs, OH^−^ ions were introduced to fulfill kinetically controlled the growth.

Figure S1 in the Supporting Information illustrates the growth process of highly and lowly uniform STNPs. The intended STNPs were centrifuged at 9600 rpm/10 minutes to remove the excessive anion and cation in the supernate. As shown in Figure S3 in the Supporting Information, when nonuniform STNPs were prepared, the full width at half maximum (fwhm) of the plasmon resonance absorption spectrum was as high as about 117 nm. However, when the OH^−^ ions were introduced into the reaction and under an effective control, the fwhm value can be decreased to be only ~80 nm, which undoubtedly revealed the highly uniform size and narrow size distribution of the resulting STNPs. The molar concentration of the STNPs was calculated based on the consideration of the density of silver as 10.3 g/cm^3^. As a result, the concentration of the prepared STNPs was calculated to be about 3 nmol/L for the next experiments.

### Synthesis of aqueous CdTe/CdS core/shell QDs

The MPA stabilized QDs were synthesized in aqueous solution referenced by the previous method^23^. The obtained red QDs were emitted at 612 nm with QY of 50%. The diameter of the QDs was calculated to be 4.5 nm according to the first exciton absorption peak referenced by Peng *et al.*^24^, which is basically in accordance with the Transmission Electron Microscopy (TEM) images (Figure S6 in the Supporting Information). The small size smoothly excludes the disadvantage of the large size (12–30 nm) water-soluble QDs after the multilayer surface coating. This is because the thinner surface capped layer will extremely enhance the FRET efficiency if QDs are acted as donors since the FRET efficiency is inversely proportional to the sixth-power of the distance between donor and acceptor^3^,^25^.

### Conjugation of STNPs and antibody (Ab)

Because the abundant citric acid groups surrounded in the surface of the STNPs^21^. Therefore, it is easy to prepare the STNPs-Ab conjugates through non-covalent adsorption, e.g., non-specific electrostatic attachment, etc.^26^. Basically, 60 pmol STNPs were mixed with 6 nmol Ab via a gentle stirring for single labelling. The adsorption time of Ab on STNPs should be sufficient (not less than 2 h) in order to immobilize the HBsAb1 sufficiently. After the non-covalent adsorption, the STNPs-Ab conjugates were blocked with addition of 1% BSA solution. After a more than 10 minutes’ centrifugation of the samples, the resulting conjugates could be collected readily.

### Conjugation of QDs and antibody

The HBsAb2 was conjugated to the QDs by using EDC and sulfo-NHS as cross-linking reagent referenced by the previous work^1^. The schematic procedure of this covalent binding is shown in Figure S5 in the Supporting Information.

### Homogeneously immune detection based on the FRET from QDs to STNPs

Higher antigen concentration HIA: as shown in Fig. 1, firstly, 30 μL BSA blocked STNPs-Ab1 (70 nM) and QDs-Ab2 (70 nM) samples were mixed in PBS (pH 7.4) solution; afterwards, the detected antigens with different amount were added into the solution for about 60 minutes’s Ag-Ab immune reactions to achieve different concentrations of 2.5, 5, 10, 20, 40, 80, 160 and 320 ng/mL for HBsAg. The detected solutions’ volume was fixed at 300 μL. The resulted mixtures were added into a 2 mm path length quartz cell for the PL measurements. The excitation slit and emission slit were set as 5 nm. The control experiments were also carried out where no antigen was added. Because the FRET induced quenching of QDs’ fluorescence is corelated with the detected antigens, we could get the immune detection curve readily.

Lower antigen concentration HIA: as shown in Fig. 1, firstly, 1.5 μL BSA blocked STNPs-Ab1 (70 nM) and QDs-Ab2 (70 nM) samples were mixed in PBS (pH 7.4) solution; afterwards, the detected antigens with different amount were added into the solution for about 60 minutes’s Ag-Ab immune reactions to achieve different concentrations of 0.1, 0.3, 0.9, 2.7, 8.1 and 24.3 ng/mL for HBsAg. The detected solutions’ volume was fixed at 300 μL. The resulted mixtures were added into a 2 mm path length quartz cell for the PL measurements. The excitation slit and emission slit were set as 10 nm. The control experiments were also carried out where no antigen was added. Because the FRET induced quenching of QDs’ fluorescence is corelated with the detected antigens, we could get the immune detection curve readily.

### Characterization

The morphology and size distribution of STNPs were characterized by Field Emission Scanning Electron Microscopy (FE-SEM, Hitachi, S-4800) and the voltage is 5 kV. The size of QDs was either determined by a TEM (JEOL-3010) measurement or calculated with the wavelength of the first exciton absorption peak^23^. Absorption and PL spectra were measured at indoor temperature by UV-3101 spectrophotometer and Hitachi F-7000 fluorescence spectrofluorimeter, respectively. The excitation wavelength for PL spectra was 400 nm.

## Additional Information

**How to cite this article**: Zeng, Q. *et al.* Highly Sensitive Homogeneous Immunoassays Based on Construction of Silver Triangular Nanoplates-Quantum Dots FRET System. *Sci. Rep.* **6**, 26534; doi: 10.1038/srep26534 (2016).

## Supplementary Material

Supporting Information
