# Supplementary material for: Highly Sensitive Homogeneous Immunoassays Based on Construction of Silver Triangular Nanoplates-Quantum Dots FRET System
Source: Sci Rep. 2016 May 20;6:26534. doi: 10.1038/srep26534 (PMC4873782; doi:10.1038/srep26534)
Supplement: Supporting Information [file srep26534-s1.doc]

**Supporting Information**

**Highly Sensitive Homogeneous Immunoassays Based on Construction of Silver Triangular Nanoplates-Quantum Dots FRET System**

Qinghui Zeng,^1^ Qin Li,^2^ Wenyu Ji,^1, *^ Bin Xue,^1^ and Jie Song^3, *^

*1 State Key Laboratory of Luminescence and Applications, Changchun Institute of Optics, Fine Mechanics and Physics, Chinese Academy of Sciences, Dong_Nanhu Road 3888, Changchun 130033, P. R. China. 2 Queensland Micro- and Nanotechnology Centre & Environmental Engineering,* [*Griffith*](mailto:song@inano.au.dk) *University, Brisbane, QLD 4111, Australia. 3 Institute of Nano Biomedicine and Engineering, Department of Instrument Science and Engineering, School of Electronic Information and Electrical Engineering, Shanghai Jiao Tong University, 800 Dongchuan Road, Shanghai 200240, PR China. Correspondence and requests for materials should be addressed to W.J. (email:* [*jiwy@ciomp.ac.cn*](mailto:jiwy@ciomp.ac.cn)*) or J.S. (npng001@gmail.com)*

**Calculation of the extinction coefficient of the STNPs**

The extinction coefficient of the STNPs is calculated according to the classical formulation: ε=A/c*l, here A is the absorption value of the STNPs’ solution which can be got from the measurement of the absorption spectrum, l is the length of the absorption cell. In our work, l is 1 cm. c is the molar concentration of the STNPs. The weight if one STNP (m) can be calculated by the followed formula: m=ρV, here ρ is the density of silver (10.3 g/cm^3^), V is the volume of one STNP, which is calculated according to the formulation: V=0.433a^2^h. Here a and h are the side length and the thickness of the STNP, respectively. According to the given silver ions’ molar concentration, the volume of the triangular nanoplates and the molecular weight of silver (107 g/mol), the concentration of STNPs is not difficult to be obtained. As a result, the extinction coefficient is easily calculated to be about 6*10^12^ M^-1^cm^-1^.


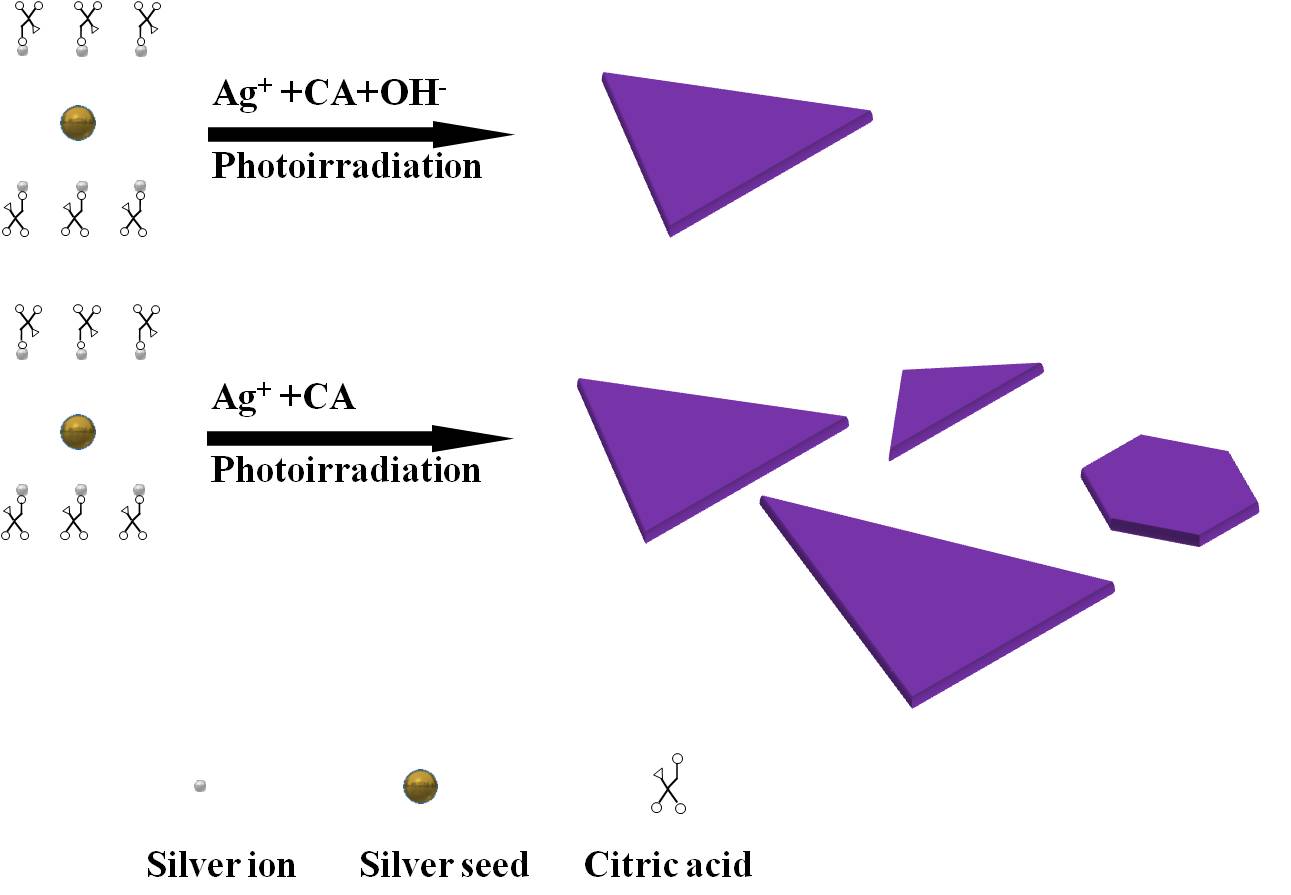


**Figure S1.** The schematic growth of highly uniform (with additional controlling of OH^-^ ions) and nonuniform STNPs (without additional controlling of OH^-^ ions).


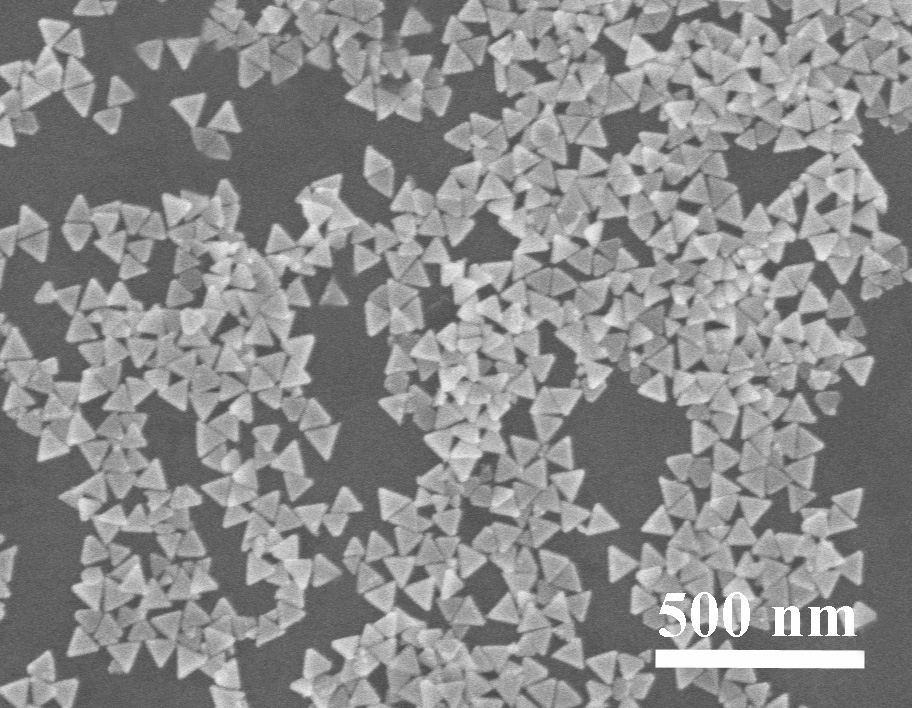

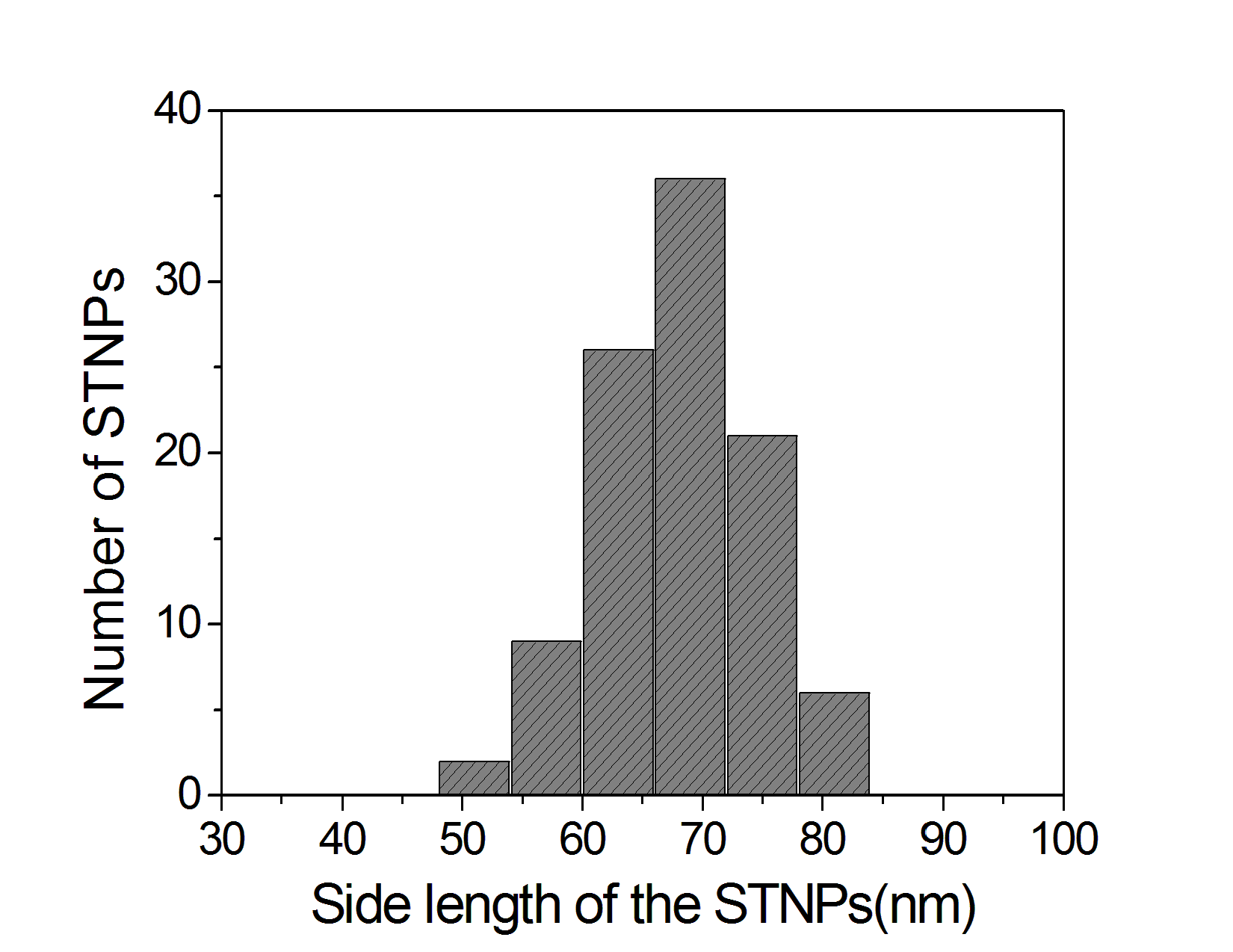


**Figure S2.** Low magnification FE-SEM images of the STNPs prepared in our method and the corresponding statistical schematics of the side length of the STNPs. The scale bar is 500 nm.


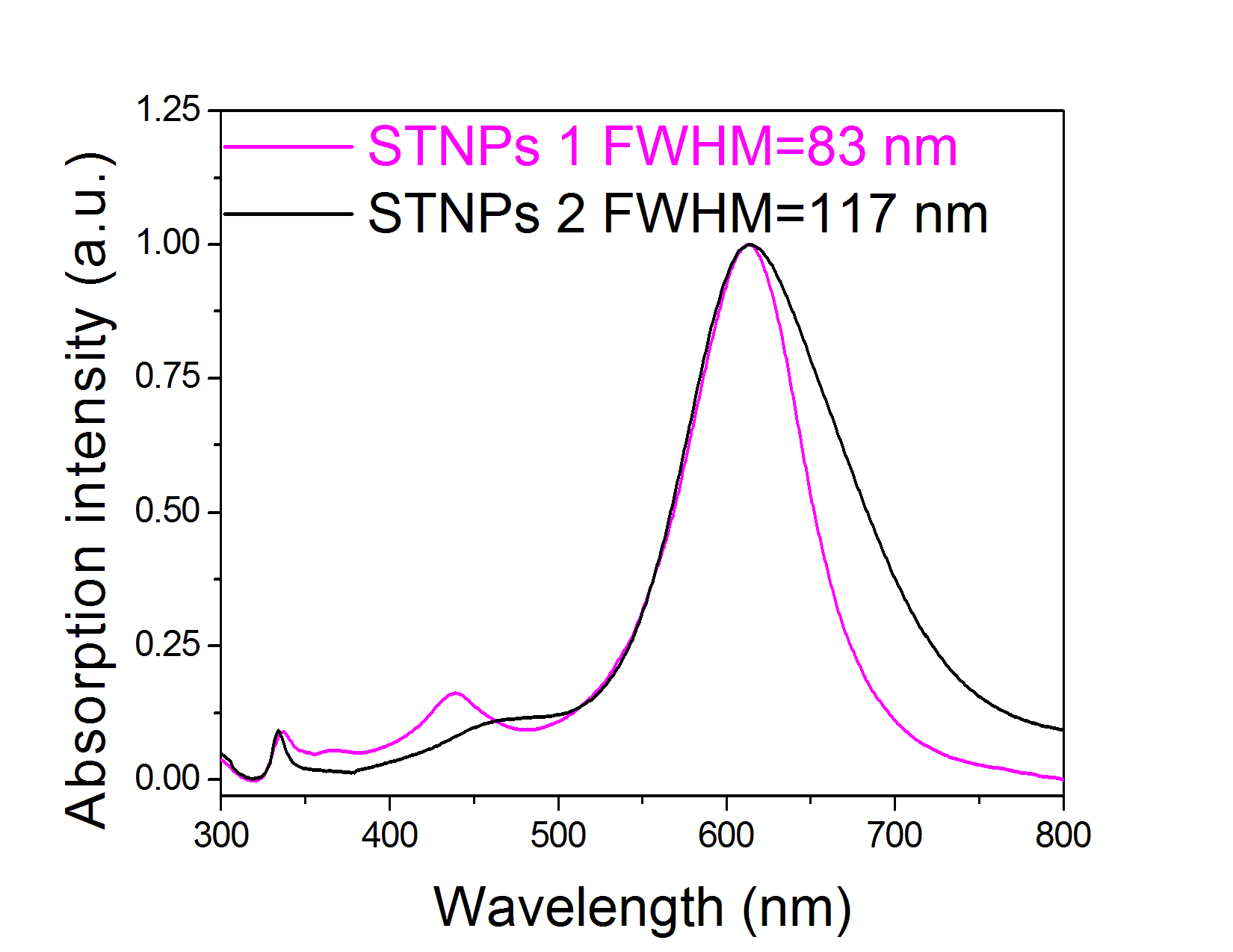

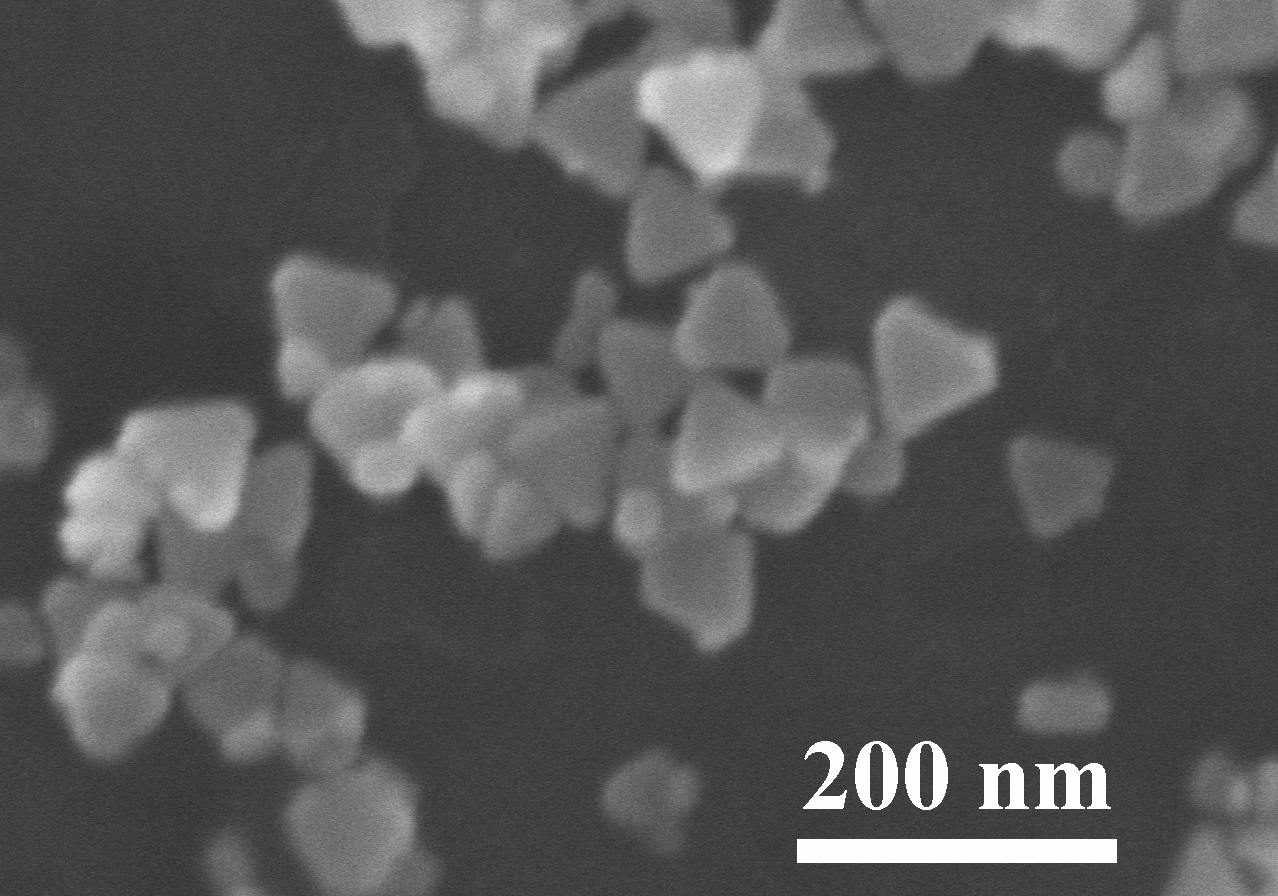


**Figure S3.** Normalized absorption spectra of highly uniform (with additional controlling of OH^-^ ions, purple curve) and nonuniform STNPs (without additional controlling of OH^-^ ions, black curve). The FE-SEM image of the nonuniform STNPs was shown in the right.


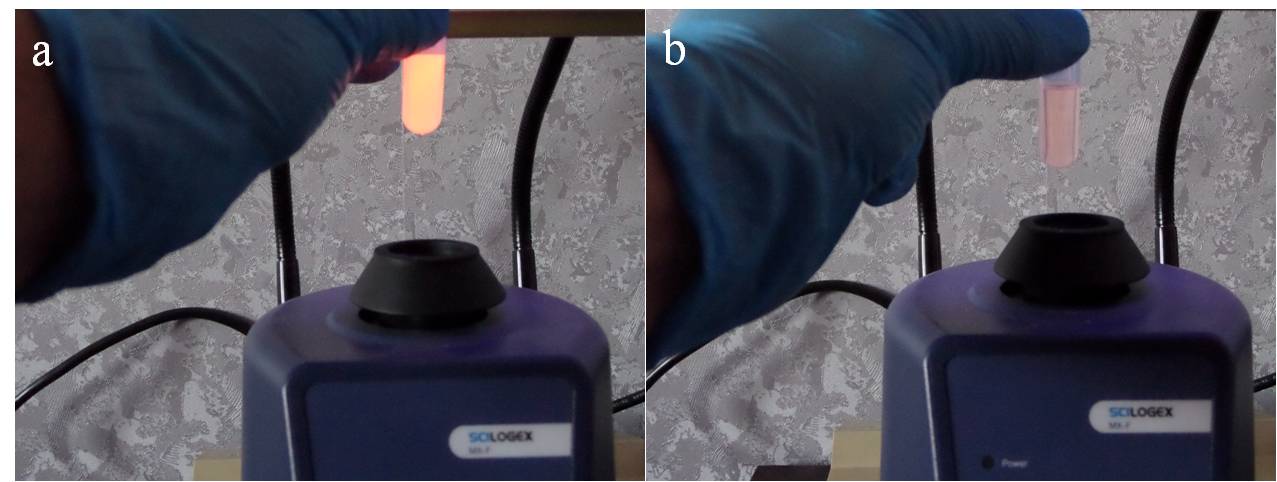


**Figure S4.** The digital photo of the QDs’ solution before (a) and after (b) the addition of ethylenediamine modified STNPs under the excitation of the ultraviolet lamp.


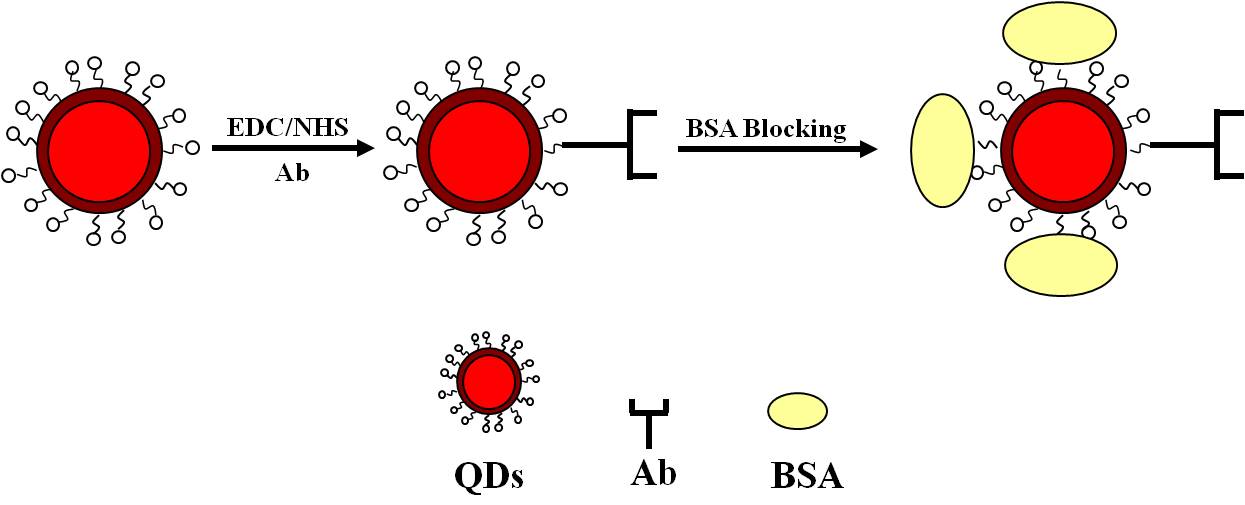


**Figure S5.** The schematic route of the covalent conjugation of QDs to HBsAb by using EDC/NHS as the condensing agents.


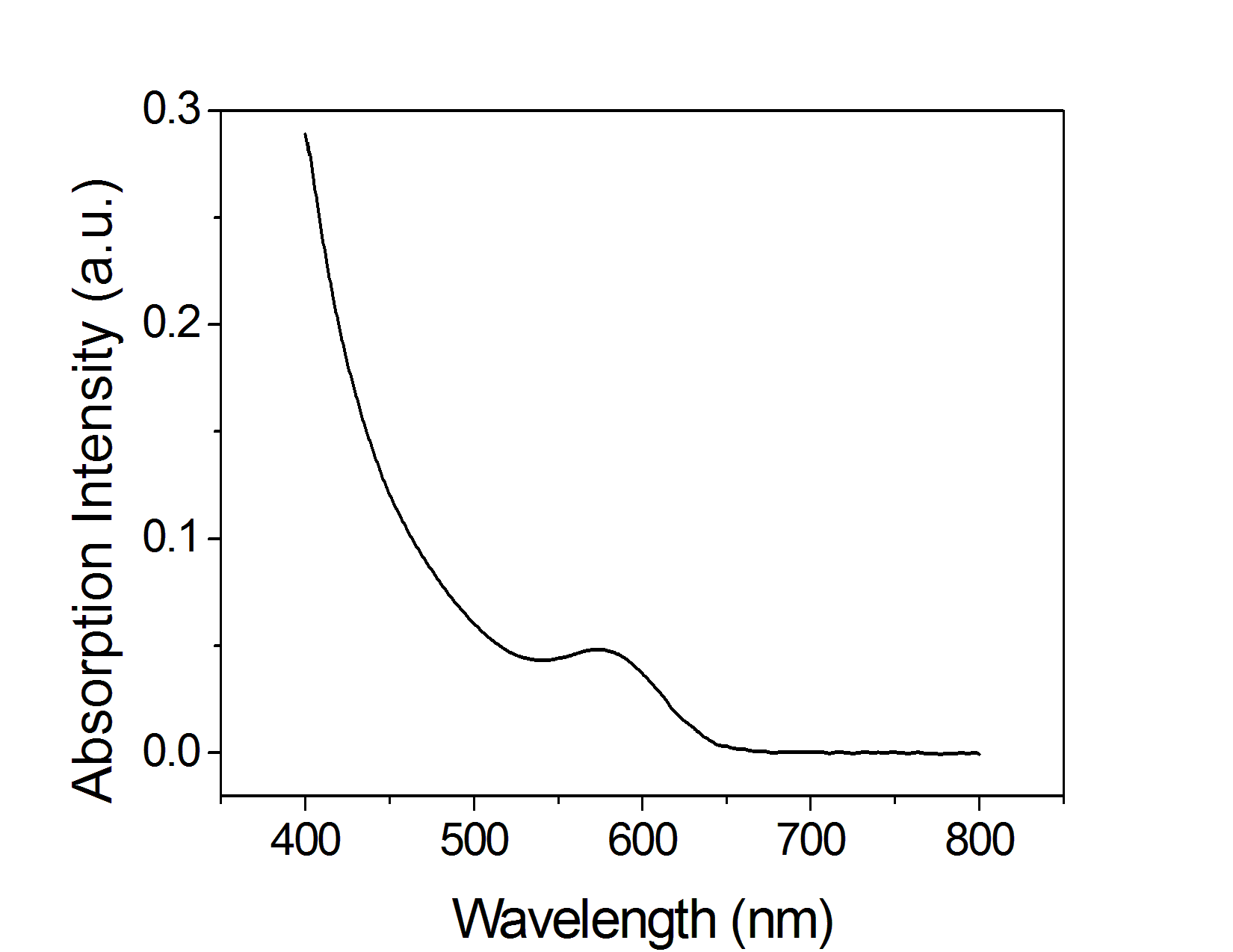

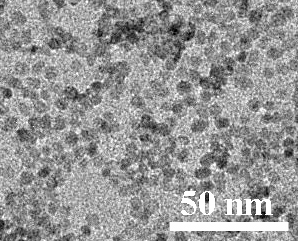


**Figure S6.** Absorption spectrum and TEM images of the QDs prepared in this work. The scale bar is 50 nm.

|  | STNPs | GNRs |
| --- | --- | --- |
| surface area/nanoparticle | ~4200 nm^2^ | ~6000 nm^2^ |
| percentage of curvature area | 0% | 100% |
| extinction coefficient | ~10^12^ M^-1^cm^-1^ | ~10^10^ M^-1^cm^-1^ |
| absorption at the excitation wavelength (400 nm) | lower | higher |
| maximum FRET efficiency | 98% | 91% |
| LOD of HBsAg detection | 0.3 ng/ml | 8.3 ng/ml |

**Table S1.** Relevant parameters displaying to show the comparison between STNPs and GNRs for explaining the reason of the higher FRET efficiency and lower LOD of the antigen detection.
